# Statement of Retraction: MicroRNA-335-5p alleviates inflammatory response, airway fibrosis, and autophagy in childhood asthma through targeted regulation of autophagy related 5

**DOI:** 10.1080/21655979.2024.2299556

**Published:** 2024-02-20

We, the Editors and Publisher of the journal *Bioengineered*, have retracted the following article:

Qingbin Liang, Jingjing He, Qian Yang, Qinghua Zhang & Yingjun Xu (2022) MicroRNA-335-5p alleviates inflammatory response, airway fibrosis, and autophagy in childhood asthma through targeted regulation of autophagy related 5, Bioengineered, 13:1, 1791-1801, DOI: 10.1080/21655979.2021.1996315

Since publication, significant concerns have been raised about the integrity of the data and reported results in the article. When approached for an explanation, the authors did not provide their original data or any necessary supporting information. As verifying the validity of published work is core to the integrity of the scholarly record, we are therefore retracting the article. All authors listed in this publication have been informed.

We have been informed in our decision-making by our policy on publishing ethics and integrity and the COPE guidelines on retractions.

The retracted article will remain online to maintain the scholarly record, but it will be digitally watermarked on each page as ‘Retracted’.

